# Scale-invariant Mexican Hat wavelet descriptor for non-rigid shape similarity measurement

**DOI:** 10.1038/s41598-023-29047-4

**Published:** 2023-02-13

**Authors:** Yuhuan Yan, Mingquan Zhou, Dan Zhang, Shengling Geng

**Affiliations:** 1 State Key lab of Tibetan Intelligent Information Processing and Application, Xining, 810008 China; 2 Qinghai Normal University, Academy of Plateau Science and Sustainability Development, Xining, 810008 China; 3 Qinghai Normal University, the School of Computer Science, Xining, 810008 China

**Keywords:** Computer science, Information technology

## Abstract

The Mexican Hat wavelet (MHW) is strictly derived from the heat kernel by taking its negative first-order derivative with respect to time *t*. As a solution to the heat equation that the heat kernel has a clear initial condition, the Laplace–Beltrami operator. Although the MHW descriptor can effectively characterize the model information, but it has poor robustness to the model with scale transformation, and the feature description performance is affected to some extent. Following a popular mathematical method, in this paper, we bases on the MHW to study scaling invariance and proposes a new shape descriptor, the scale-invariant Mexican Hat wavelet (SIMHW), which by logarithmic sampling and Fourier transform that obtains the expression of SIMHW in Fourier domain. The experimental results show that SIMHW has finer information description ability and stronger recognition ability, and has better robustness to various non-rigid transformations. It can correctly calculate the similarity between 3D shapes and realize the effective shape retrieval.

## Introduction

With the improvement of 3D shape generation technology, 3D models have been developed in many fields, such as industrial production, molecular biology, medical research, mechanical engineering and heritage conservation. Driven by these applications, various shape analysis tasks that used 3D shapes as input have been developed, including shape matching^1^, object recognition and segmentation^2^, point to point correspondence^3^, shape reconstruction^4^ and shape retrieval^5^. In many shape analysis tasks, the most fundamental and important problem that is how to design a reasonable and effective shape representation method^6^. At present, many shape descriptors that can be used to identify and analyze models with different transformation poses(i.e. translation, scaling, rotation, isometric transformation, etc.) have been proposed^7^. The early research on 3D shape descriptors mainly focused on the invariance of rigid transformations(such as Euclidean transformation), and more classical methods such as, shape distributions^8^, projection views^9^, spherical harmonic transformations^10^, topological transformations^11^ and geometric moments^12^.

However, when dealing with real 3D data, most shape analysis problems are based on non-rigid transformations, and the most typical is isometric transformation that can change the shape surface information without tearing or stretching, which brings great challenge to shape analysis. In the past decade, the method of diffusion geometry has made great progress in 3D shape analysis, which the idea behind these methods was first proposed by Coifman and Lafon^13^ in 2006. These approaches leverage the relation between the geometry of the underlying space and the diffusion process defined on it, as encoded especially by the spectrum of the Laplace–Beltrami (LB) operator^14^, which mainly solves the problem of isometric or near-isometric transformations. Among many studies, the method based on spectral analysis^15^ has quickly become a new research hotspot due to its isometric invariance. Since the LB operator only depends on the intrinsic properties of shape, which makes the spectral shape descriptor is very suitable for finding similar mappings between isometric or near-isometric shapes, and comparing the same object with different expressions or poses. By far, the most point signatures^16^ and spectrum shape analysis^17^ approaches use the eigenvalues and eigenfunctions of the LB operator, which is a natural extension of the Fourier basis on surfaces.

Reuter^18^ proposed a descriptor called ‘Shape-DNA’, which used truncated spectra as a global feature. Similar to this idea, the global point signature (GPS) was introduced by Ovsjanikov^19^. The GPS descriptor has good performance under isometric transformation, but it will be affected by such factors as symbol inversion and change of eigenfunction sequence. According to the basic solution of the heat equation, Sun^16^ proposed a more reliable local point signature descriptor, the heat kernel signature(HKS). This signature has high computational efficiency and is robust to small shape disturbances and near-isometric transformations, but its performance is affected by the time parameter. To address the poor feature localization of HKS, Aubry^20^ introduced the wave kernel signature (WKS) and used a different physical model (i.e.,Schrödinger equation) to replace heat diffusion, and then analyzed the behavior of quantum particles on the shape surface. However, the above shape descriptors are very sensitive to scale transformation. To eliminate the scaling factor, based on HKS, Brosten^21^ proposed the scale-invariant heat kernel signature(SIHKS) by using a Fourier transform. Li^22^ established a scale-invariant wave kernel signature (SIWKS) to process the scale-transformed 3D shape retrieval task. Du^23^ combined HKS features with curvature information to eliminate the influence of time parameters, and extracted the points with the largest change in HKS values as the feature points of the model. With the increasing availability of textured models, color features are becoming more and more important in 3D shape analysis. Combined with color information, Biasotti^24^ analyzed and evaluated spectral geometry-based methods for the retrieval and classification of textured 3D models. Based on the missing parts non-rigid 3D dataset, Masoumi^25^ compared and evaluated the retrieval performance of low-level local features, middle-level local features and high-level aggregated feature descriptors constructed by local spectral descriptors. Zhang^26^ provided an effective measurement of the 3D skull similarity harmonic wave kernel signature (HWKS), which can effectively be used to extract geometric and topological information. Compared with the wave kernel feature, the local and global skull information can be described at the same time using this signature.

Wavelet transform is another important tool for shape analysis and model processing, which can provide better localization information in the space or frequency domain, but it is never easy to define wavelets on manifolds. In recent research results, mathematicians have studied generating wavelets through the use of spectral theory. Hammond^27^ addressed graph wavelets through spectral graph theory. The graph wavelets are generated by a wavelet operator expanded on eigenfunctions of the graph Laplacian. Antoine^28^ also studied continuous wavelet transforms on graphs, constructed by a generator in the spectral domain. As an example, they introduced the Mexican Hat wavelet (MHW), which is the Fourier transform of the Euclidean MHW, that was formulated by the generator. Hou^29^ systematically studied the well-known MHW in manifold geometry, including its derivation, properties, transforms, and applications. Masoumi^30^ proposed a spectral graph wavelet signature (SGWS) approach for 3D shape classification using the bag-of-features (BOF) paradigm, which the local descriptors are extracted via the spectral graph wavelet transform having the MHW as a generating kernel. In addition, Masoumi^31^ also presented a deep learning approach to 3D shape classification using SGWS and the BOF paradigm. This approach not only captures the similarity between feature descriptors via a geodesic exponential kernel, but also substantially outperforms state-of-the-art methods both in classification accuracy and in scalability. Kirgo^32^ proposed a new construction for MHW on shapes with applications to partial shape matching. This novel construction method used the derivative of the approximate heat kernel to rapidly compute a multiscale family of MHW functions.

Different from the Euclidean metric, it is very difficult to define a function explicitly on a manifold. Many existing methods rely on differential geometry, in which surface parameterization is often unavoidable. In this work, we study the well-known MHW in manifold geometry. Firstly, based on the diffusion wavelet, we propose to construct a multiscale MHW function by the negative first-order derivative of the heat kernel. In the Euclidean domain, this time derivative(or the equivalent second derivative in space) corresponds to the MHW. Then, inspired by the characteristics of scale-invariant heat kernel signature (SIHKS), we discuss the scaling invariance of MHW, and strictly derive the explicit expression of the scale-invariant Mexican Hat wavelet (SIMHW) in Fourier domain, which provides a very effective and powerful method for local shape analysis.

The paper mainly studies some important properties of SIMHW, and further discusses the performance of shape descriptor and the effect of similarity calculation. The main contributions can be summarized as follows:

- We propose the SIMHW descriptor as a new tool for localized shape analysis, which can be used to more effectively describe the local detail difference of the shape; It has the invariance of scale and isometric transformations, and has good robustness to topology, noise and sampling transformations.
- We propose to use the modified Hausdorff distance (MHD) calculation method to measure the matching degree between 3D shapes, which has obvious competitiveness in shape similarity comparison and shape retrieval.
- The spectral shape descriptor SIMHW has strong extensibility and scalability, which can be transferred to other differential operators on the manifold for shape analysis, and can also be used to analyze point cloud shape data.

## Methods

We take the negative first-order derivative of the heat kernel with respect to time to represent MHW accurately, which is compact and computationally efficient. Although the obtained MHW descriptor has more powerful analysis ability than HKS, but, it is very sensitive to scale transformation. In view of the construction process of SIHKS, we defined a scale-invariant Mexican hat wavelet (SIMHW) descriptor, which is invariant to scale transformation and has better shape analysis capability.

### Laplace–Beltrami operator discretization

The LB operator can be regarded as one of the most fundamental operators in image processing and shape analysis^33^. For triangular mesh shapes *M*, the cotangent weight scheme^34^ is generally used to approximate the discretized LB operator. The LB operator at vertex $v_{i}$ can be expressed as:

1 $$\begin{aligned} {\bigtriangleup }_{M} f(v_{i}) \approx \frac{1}{a_{i}}{\sum \limits _{i,j}}w_{ij}( f(v_{i})-f(v_{j}) ) \end{aligned}$$

where $a_{i}$ is the area of the Voronoi cell at vertex $v_{i}$, and $w_{ij}={(\cot \alpha _{ij}+\cot \beta _{ij})}/{2}$ represented cotangent weight, $v_{j}$ is in the 1-ring neighborhood of vertex $v_{i}$, $\alpha _{ij}$ and $\beta _{ij}$ are the angles $<v_{i}v_{j+1}v_{j}$ and $<v_{i}v_{j-1}v_{j}$ of two faces $f^{\alpha }={v_{i},v_{j},v_{j+1}}$ and $f^{\beta }={v_{i},v_{j},v_{j-1}}$ that are adjacent to the edge $e_{ij}$. It is worth pointing out that the cotangent weight scheme retains several important properties of continuous LB operator, such as symmetry and positive semi-definiteness. The first $k\le n$ eigenfunctions and eigenvalues of the LB operator are computed by performing the generalized spectral decomposition: ${\textbf {W}}\varvec{\varphi }={\textbf {A}}\varvec{\varphi }\varvec{\varLambda }$, here $\varvec{\varLambda }=(\lambda _{1},\lambda _{2}, \ldots ,\lambda _{k})$ is the diagonal matrix of eigenvalues, and $\varvec{\varphi }=(\varphi _{1},\varphi _{2}, \ldots ,\varphi _{k})$ is a discretized eigenfunctions matrix. It is noteworthy that the eigensystem $\{\lambda _{i},\varphi _{i}\}$ is an intrinsic property of the manifold and has isometric invariance, which is very suitable for analyzing non-rigid 3D shapes. The lower eigenvalues associated with eigenfunctions represent a low frequency information of the shapes, while the higher eigenvalues correspond to a high frequency information that can describe the details of the shapes. Based on the obtained eigenfunctions and eigenvalues, many studies have proposed to use spectral signatures to describe the vertex features of the shape surface.

### Mexican Hat wavelet descriptor

Let *M* be a compact Riemannian manifold possibly with boundary. The heat diffusion process over *M* is governed by the heat equation:

2 $$\begin{aligned} {\bigtriangleup} _{M}{u(x, t)}=-\frac{\partial u(x, t)}{\partial t} \end{aligned}$$

where $\bigtriangleup _{M}$ is the LB operator of *M*, it is well-known that for any *M*, there exists a function $k_{t}(x, y):R^{+}\times M\times M\longrightarrow R$ such that $u(x, t)=\int _{M}k_{t}(x, y)f(y)dy$, the *dy* is the volume form at $y\epsilon M$, the function $k_{t}(x, y)$ is called heat kernel, and can be thought of as the amount of heat that is transferred from *x* to *y* in time *t* given a unit heat source at *x*. For compact manifold *M*, the heat kernel has the following eigendecomposition: $k_{t}(x, y)={\sum \nolimits _{i=0}^\infty e^{-\lambda _{i}{t}}{\varphi _{i}(x)}{\varphi _{i}(y)}}$. $\lambda _{i}$ and $\varphi _{i}$ are the $i^{th}$ eigenvalue and the *i*th eigenfunction of the LB operator, respectively. To reduce the computational complexity, researchers limited the heat kernel to a certain time domain and defined a more concise local shape descriptor, the heat kernel signature (HKS):$h_{t}(x, x)=k_{t}(x, x)={\sum \nolimits _{i=0}^\infty e^{-\lambda _{i}{t}}{\varphi _{i}^{2}(x)}}$.

The heat kernel in the Fourier domain $\hat{h}_{t}(i)$ is a Gaussian of $\sqrt{\lambda _{i}}$. This implies that, although the heat kernel has no closed-form expression in space, but it has an explicit expression as a Gaussian in the Fourier domain:

3 $$\begin{aligned} \left\{ \begin{array}{c} h_{t}(x, x)={\sum \nolimits _{i=0}^\infty e^{-\lambda _{i}{t}}{\varphi _{i}^{2}(x)}}\\ \hat{h}_{t}(i)= e^{-\lambda _{i}{t}} \end{array} \right. \end{aligned}$$

The Euclidean MHW is defined as the negative first-order derivative with respect to time *t* (or, equivalently, the second-order derivative with respect to *x*) of the Gaussian. Cause the heat kernel in the Fourier domain $\hat{h}_{t}(i)$ is a Gaussian of $\sqrt{\lambda _{i}}$, so we can define the MHW descriptor $\psi _{t}(x,y):R^{+}\times M\times M\longrightarrow R$ on manifold geometry as the negative first-order derivative of the heat kernel $h_{t}(x,y)$. At a given scale, the negative first-order derivative of such a function constitutes the diffusion MHW, equivalently, the $\psi _{t}(x,y)$ at scale *t* can be computed from the heat kernel $h_{t}(x,y)$:

4 $$\begin{aligned} \psi _{t}(x,y)=-\partial h_{t}(x,y)={\bigtriangleup} _{x}h_{t}(x,y) \end{aligned}$$

$\bigtriangleup _{x}$ denotes the LB operator with respect to point *x*. Given the LB eigensystem $\{{\lambda _{k},\varphi _{k}}\}$, the $\psi _{t}(x,y)$ of the heat kernel in the continuous setting are given by:

5 $$\begin{aligned} \psi _{t}(x,y)=\frac{\partial }{\partial t}{h_{t}(x,y)}={\sum \limits _{i=0}^\infty \lambda _{i}e^{{-\lambda _{i}}{t}}}{\varphi _{i}(x)}{\varphi _{i}(y)} \end{aligned}$$

The MHW on shape *M* is defined as a truncated version up to the LB operator eigenpairs($i=k$), which the MHW on point *x* can be approximate representation as:

6 $$\begin{aligned} \psi (x,t)={\sum \limits _{i=0}^k\lambda _{i}e^{-\lambda _{i}{t}}}{\varphi _{i}^{2}(x)} \end{aligned}$$

### Scale-invariant Mexican Hat wavelet descriptor

A notable disadvantage of MHW is its sensitivity to scale transformation. In order to solve this problem, we define a scale-invariant Mexican Hat wavelet descriptor (SIMHW), which has the invariance of scale transformation and more efficient recognition ability. Given a shape *M*, and its scaled version(scaling factor is $\beta$) can be expressed as $M^{\prime }=\beta M$. According to the spectral decomposition of the LB operator, the new eigenvalues and eigenfunctions will satisfy $\lambda ^{\prime }=\beta ^{2}\lambda$ and $\varphi ^{\prime }=\beta \varphi$. Therefore, the MHW descriptor that contains scale information can be expressed as:

7 $$\begin{aligned} \psi ^{\prime }(x,t)=\sum \limits _{i=0}^k\beta ^{2}\lambda _{i} e^{-\beta ^{2}\lambda _{i}{t}}{\varphi _{i}^{2}(x)}{\beta ^{2}}={\beta ^{4}}{\sum \limits _{i=0}^k\lambda _{i}e^{-\lambda _{i}{\beta ^{2}}{t}}{\varphi _{i}^{2}(x)}}={\beta ^{4}}\psi (x,{\beta ^{2}t}) \end{aligned}$$

In order to achieve the invariance of scale transformation, we need to remove the scaling factor $\beta$ from $\psi$. Firstly, the time of MHW at any point $x\epsilon M$ is transformed to $t=\alpha ^{\tau }$ that we can get $\beta ^{2}{t}=\beta ^{2}\alpha ^{\tau }$, so the function $\psi ^{\prime }(x,t)$ can be expressed as $\psi ^{\prime }(x,t)={\beta ^{4}}\psi (x,{\beta ^{2}\alpha ^{\tau }})$. Then the logarithmic transformation of the base of $\alpha$ is performed on the formula $\beta ^{2}\alpha ^{\tau }$, and we can obtain $\log _{\alpha }\beta ^{2}\alpha ^{\tau }={\tau }+2\log _{\alpha }{\beta }$. So the MHW of the scaled shape $M^{\prime }$ becomes $\psi ^{\prime }(x,{\tau })=\beta ^{4}\psi (x,\tau +2log_{\alpha }{\beta })$. Finally, the constant factor $\beta ^{4}$ can be removed by taking the logarithm of the $\psi ^{\prime }(x,\tau )$ and then solving the first-order differential of $\tau$:

8 $$\begin{aligned} \log \psi ^{\prime }(x,{\tau })={4\log \beta +\log \psi (x,\tau +2\log _{\alpha }\beta )}, \frac{d}{d\tau }{\log \psi ^{\prime }(x,{\tau })}=\frac{\frac{d}{d\tau }{\psi (x,\tau +2\log _{\alpha }\beta )}}{{\psi (x,\tau +2\log _{\alpha }\beta )}} \end{aligned}$$

If the MHW with the constant factor $\beta ^{4}$ eliminated is expressed as $\psi ^{*}(x,{\tau })$, then:

9 $$\begin{aligned} \psi ^{*}(x,{\tau })=\frac{{\frac{d}{d\tau }{\psi (x,\tau )}}}{\psi (x,{\tau })}=\frac{-\sum \limits _{i=0}^k\lambda _{i}^{2}{\log \alpha }\alpha ^{\tau }{e^{-\lambda _{i}\alpha ^{\tau }}}{\varphi _{i}^{2}(x)}}{\sum \limits _{i=0}^k\lambda _{i}{e^{-\lambda _{i}\alpha ^{\tau }}}{\varphi _{i}^{2}(x)}} \end{aligned}$$

A new function $\tilde{\psi }$ with scale transformation on time scale $\tau$ is obtained: ${\tilde{\psi }}^{*}(x,{\tau })={\tilde{\psi }}(x,\tau +2log_{\alpha }{\beta })$, here $\psi ^{*}(x,{\tau })=\psi (x,\tau +2log_{\alpha }{\beta })$. Through studying the previous literature, we can know that the Fourier transform can convert the scale transformation in time domain to the translation transformation in frequency domain. Therefore, the discrete-time Fourier transform of ${\tilde{\psi }}^{*}(x,{\tau })$ converted the shift in time of MHW into a complex phase transformation: ${\tilde{\psi }}^{*}(x,\omega )=\tilde{\psi }(x,\omega )e^{-i\omega 2log_{\alpha }{\beta }}$, here $\omega \epsilon [0,2\pi ]$ indicates the frequency domain transformation range, ${\tilde{\varPsi }}^{*}$ and ${\varPsi }^{*}$ denote the discrete-time Fourier transform of ${\tilde{\psi }}^{*}$ and ${\psi }^{*}$, respectively. The phase transformation is in turn eliminated by taking the Fourier transform modulus(FTM):

10 $$\begin{aligned} \left\{ \begin{array}{c} F[{\tilde{\psi }}^{*}(x,{\tau })](\omega )={\tilde{\varPsi }}^{*}(x,\omega )={\tilde{\varPsi }}(x,\omega )e^{-i\omega 2log_{\alpha }{\beta }}\\ \arrowvert {\tilde{\varPsi }}^{*}(x,\omega )\arrowvert =\arrowvert {\tilde{\varPsi }}(x,\omega )\arrowvert \end{array} \right. \end{aligned}$$

Here $F[{\tilde{\psi }}^{*}(x,{\tau })](\omega )$ represents Fourier transform on ${\tilde{\psi }}^{*}(x,\tau )$. We have constructed SIMHW from MHW at each point *x* that is insensitive to scale transformation. Thus, the SIMHW descriptor at point *x* on shape *M* is an n-dimensional feature vector related to the frequency component $\{\omega _{j},j=1,2, \ldots ,n\}$, which can be defined as: $SIMHW(x)=(\arrowvert {\tilde{\varPsi }}(x,\omega _{1})\arrowvert ,\arrowvert {\tilde{\varPsi }}(x,\omega _{2})\arrowvert , \ldots ,\arrowvert {\tilde{\varPsi }}(x,\omega _{n})\arrowvert )$. For the entire shape *M*, $SIMHW_{M}$ is a matrix of $m\times n$, where each row represents the n-dimensional feature vector at point $x_{i}$, and each column represents the value of all points $\{x_{i},i=1,2, \ldots ,m\}$ under frequency component $\omega _{j}$, which can be expressed as: $SIMHW_{M}=(\arrowvert {\tilde{\varPsi }}(x_{1})\arrowvert ,\arrowvert {\tilde{\varPsi }}(x_{2})\arrowvert , \ldots ,\arrowvert {\tilde{\varPsi }}(x_{m})\arrowvert )^{T}$.

**Figure 1:**
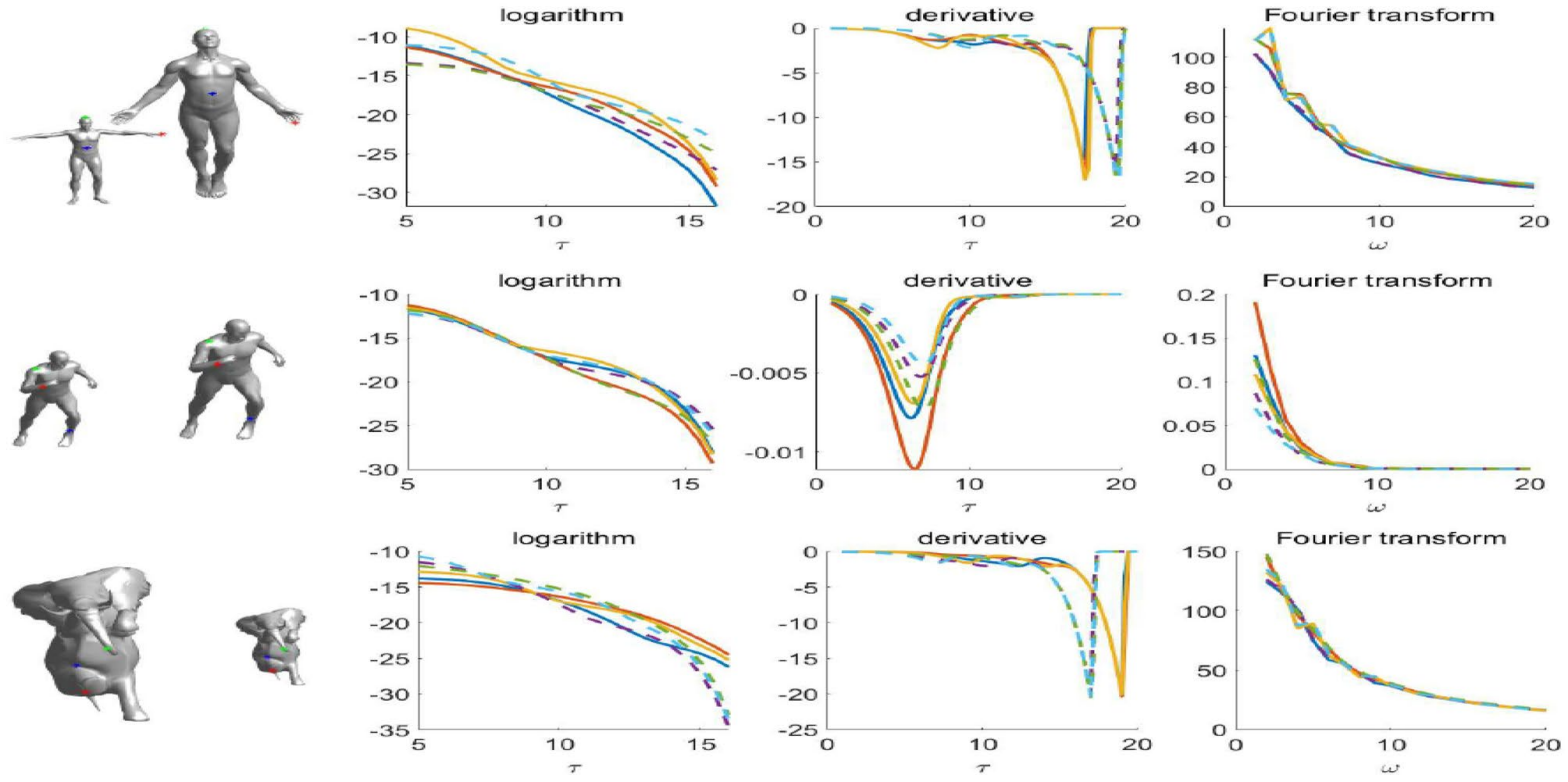
Construction results of the SIMHW descriptor: the point curve corresponding to the logarithm, derivative, and Fourier transform construction process from MHW to SIMHW.

Figure 1 shown the logarithm, derivative, and Fourier transform construction process from MHW to SIMHW, where the first column is three groups of original and scaled models(each group of models selected three pairs of corresponding points, marked with blue, green and red points respectively). The second column is the result of MHW computed at a corresponding point on a shape and its scaled version that using the scaling factor. These results plotted on a logarithmic scale, where $\psi$ and $\psi ^{\prime }$ differed by the scale and shift of $\tau$. In the third column, $\psi$ and $\psi ^{\prime }$ are shown in a case where the multiplicative constant is undone and the change in scale corresponds to a shift in $\tau$ only. In the fourth column, the frequencies of $\arrowvert \tilde{\varPsi }(x,\omega )\arrowvert$ are used as the SIMHW feature. As can be seen from the figure, the SIMHW descriptor computed at the two different scales are virtually identical.

Cause the MHW descriptor only depends on the eigenfunctions and eigenvalues of the LB operator, at least in theory, it can be used for non-rigid shape comparisons. The SIMHW descriptor also has the properties of the MHW method, which has isometric invariance, robustness to sampling, noise and topology transformations. Based on the research in this paper, it also has the invariance of scale transformation. The properties of SIMHW make it very suitable for non-rigid shape analysis, especially for shape similarity computation and shape retrieval performance analysis.

### Shape similarity measurement

There are many methods to calculate the distance between models. It is usually necessary to choose the appropriate distance measurement method according to the actual problem, and measure the similarity between the models by constructing the measurement space.

In this paper, the SIMHW features of all sampling points on the non-rigid shape are extracted to form the feature vectors of the model. Cause the sampling points between different shapes are not exactly the same, it bring great challenges to the computation of feature vectors. Based on this, we used the modified Hausdorff distance(MHD)^35^ to calculate the distance between features. When using this method, the features do not need to be aligned in advance, which compensates for the difficulty caused by different dimensions of shape features.

Marie^36^ studied 24 Hausdorff distances between two point sets and found the optimal MHD through experiments. When calculating *d*(*A*, *B*), the average of the minimum distance between each point in set $A(a\epsilon A)$ and set $B(b\epsilon B)$ is used to replace the single maximum and minimum distance, and the calculation of *d*(*B*, *A*) is same. Therefore, the influence of abnormal points on the distance calculation is weakened, and the accuracy of the distance calculation is improved, MHD is very suitable for the 3D shape similarity calculation. The pair of 3D shapes *M* and *N* based on SIMHW can be computed as:

11 $$\begin{aligned} \left\{ \begin{array}{c} MHD(M,N)=\max (d(M,N),d(N,M))\\ d(M,N)=\max \limits _{a\epsilon M}(\min \limits _{b\epsilon N}(\Vert d_{SIMHW}(M)-d_{SIMHW}(N)\Vert ))\\ d(N,M)=\max \limits _{b\epsilon N}(\min \limits _{a\epsilon M}(\Vert d_{SIMHW}(N)-d_{SIMHW}(M)\Vert )) \end{array} \right. \end{aligned}$$

Based on SIMHW for a pair of 3D shapes *M* and *N*, the MHD measured the maximum dissimilarity of the point set and is used to calculate the maximum orientation distance *d*(*M*, *N*) and *d*(*N*, *M*). If the value of *MHD*(*M*, *N*) is smaller, *M* and *N* are more matched, and vice versa. If *MHD*(*M*, *N*) is equal to zero, the 3D models *M* and *N* are completely matched.

## Results

The algorithm is implemented on a 64-bit 32GB memory Windows 10 system, using Matlab 2019b as a visual development environment. The TOSCA high-resolution dataset^37^, SHREC2010 dataset^38^ and SHREC2015-Canonical Forms dataset^39^ are used for non-rigid 3D shape analysis experiments. The TOSCA high-resolution dataset contains 80 shapes with different isometric transformation postures and is divided into 9 categories, which including cats (11), dogs (9), wolves (3), horses (8), centaur (6), gorillas (4), victoria (12) and michael and david (7 and 20, respectively). It is mainly used to verify the robustness and effectiveness of the isometric deformation models. The SHREC2010 dataset including 12 shapes, each of shape that has 12 different non-rigid deformation postures, and each deformation posture is divided into 5 different intensities. The SHREC2015 standard database including a training set and test set. The two datasets contain 10 different class of shapes, and each shape has 10 different non-rigid deformation postures, including isometric, near-isometric, topological, hole and scale transformations. These two datasets are mainly used to verify the feature description ability and shape retrieval performance of SIMHW descriptor with different non-rigid transformation models.

### Parameter settings for SIMHW descriptor

**Figure 2:**
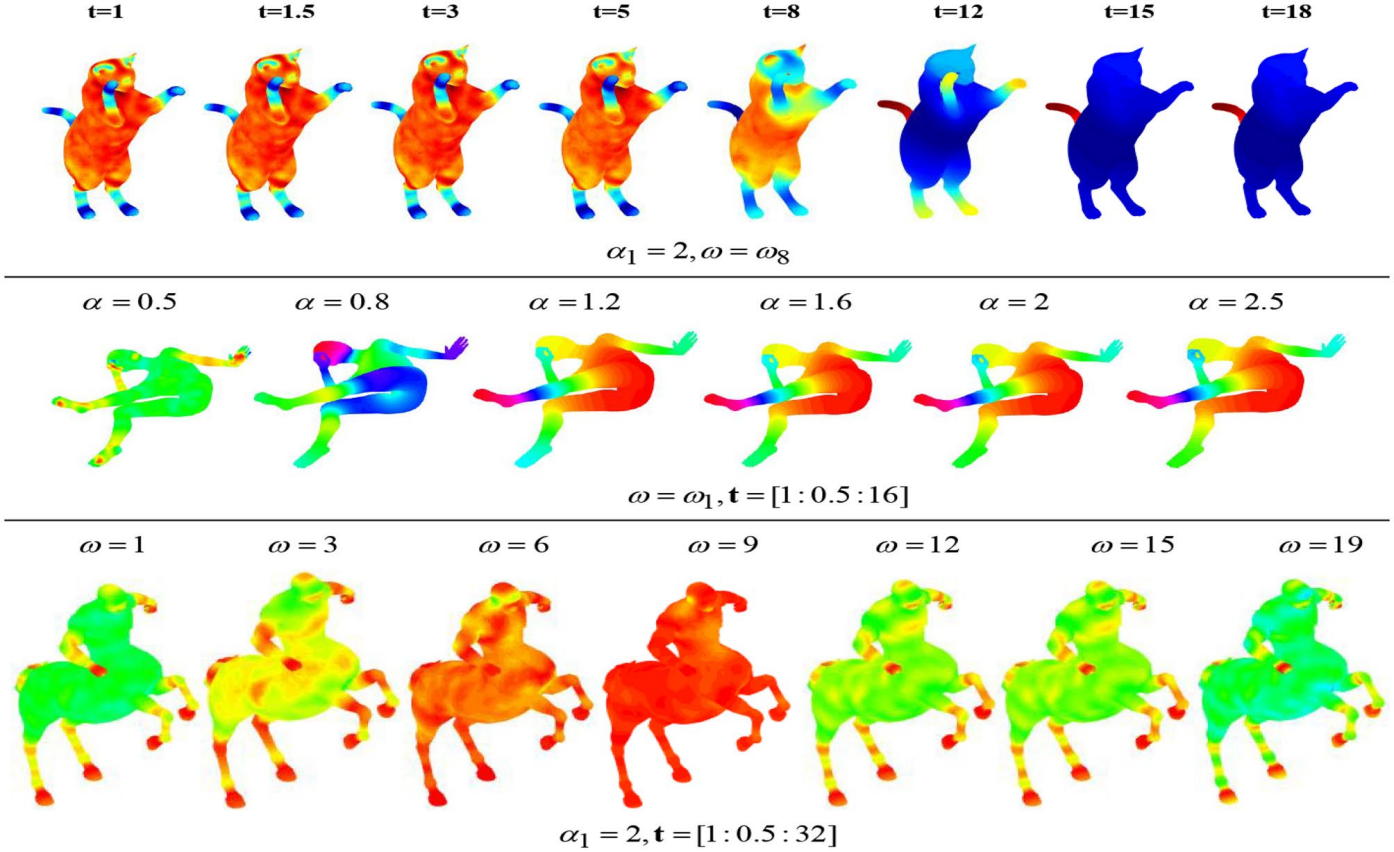
Parameter comparison for the SIMHW descriptor.

The feature thermodynamic diagrams of each parameter change of the SIMHW descriptor is shown in Fig. 2. The first row gives the multi-scale properties of the SIMHW descriptor. When the $\alpha$ and $\omega$ were given, which means that small values of *t*, the function of SIMHW is mainly determined by small neighborhoods of *x*, and these neighborhoods grow bigger as *t* increases. This implies, in particular, that for small *t*, SIMHW only reflects the local properties of the shape around *x*(the shape of the foot and tail regions are more prominent), while for large values of *t*, SIMHW captures the global structure of *M* from the point of view of *x*.

For a logarithmic scale-space with base $\alpha$, as shown in Fig. 2, the effect of the SIMHW descriptor was not significant when $\omega$ and *t* were given, for ease of calculation and comparison, we chose the same parameter settings $\alpha =2$ as in papers^16,21^. For SIMHW, which the $\omega \epsilon [0,2\pi ]$ represents the frequency range in Fourier transform modulus, and $\omega _{j}$ indicates that the *j-*th discrete lowest frequencies were used as the local descriptor. The third cow of Fig. 2 shown the SIMHW feature of different feature components $\omega _{j}$ under certain conditions of $\alpha =2$ and $t=[1:0.5:32]$. It can be seen from the figure that the description of SIMHW by different components has different characteristics. Such as, the $\omega _{1}$ component focused on the local information of the limbs of cat, while the $\omega _{6}$ or $\omega _{12}$ component can describe the global information of the shape. Cause the differences between different feature components are obvious, when SIMHW is used to describe the shapes, we also need to select one of its feature components ${\tilde{\varPsi }}(x,\omega _{i})$ or first *k* feature components ${\tilde{\varPsi }}(x,\omega _{i}),{\tilde{\varPsi }}(x,\omega _{i+1}),...,{\tilde{\varPsi }}(x,\omega _{j})$ according to the specific shape analysis problem.

MHW and SIMHW descriptors were used as local shape descriptors, respectively. For the discrete computation of the $\psi _{t}(x,.)$, we used the cotangent weight approximation of the LB operator and $k=100$. In order to compare with other spectral operators, we set $\alpha =2$ and $t=[1:0.5:16]$. After applying the logarithm, derivative, and Fourier transform, the twelfth discrete lowest frequencies $\arrowvert {\tilde{\varPsi }}(x,\omega _{12})\arrowvert$ is used as the local descriptor, which were experimentally found to give optimal performance on the database.

### Effective of the SIMHW descriptor

**Figure 3:**
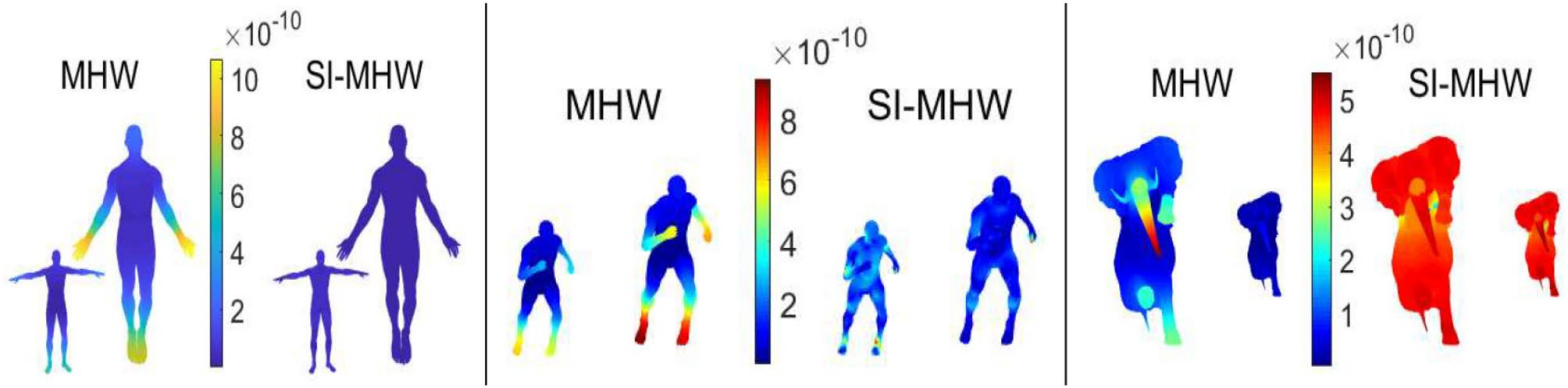
Comparison of MHW (left) and the proposed SIMHW (right) descriptors.

As shown in Fig. 3, the MHW and SIMHW thermodynamic diagrams of the three models are given, including the shapes before and after scale transformation. We construct a colormap, based on the SIMHW feature vector, we have *N* vertices and *N* color values corresponding with the *N* values of SIMHW, each value of $SIMHW_{i}$ controls each value of vertex $v_{i}$. It can be seen that MHW is basically not robust to scale transformation. For instance, the color values of the hands and feet of the human body, the feet and nose of the elephant change significantly, indicating that the shape of the same part has different MHW values; When the SIMHW descriptor is used to describe the shape, the influence of the scaling factor is eliminated, and the same region before and after the model scale transformation basically maintains the same color and has the same SIMHW value.

**Figure 4:**
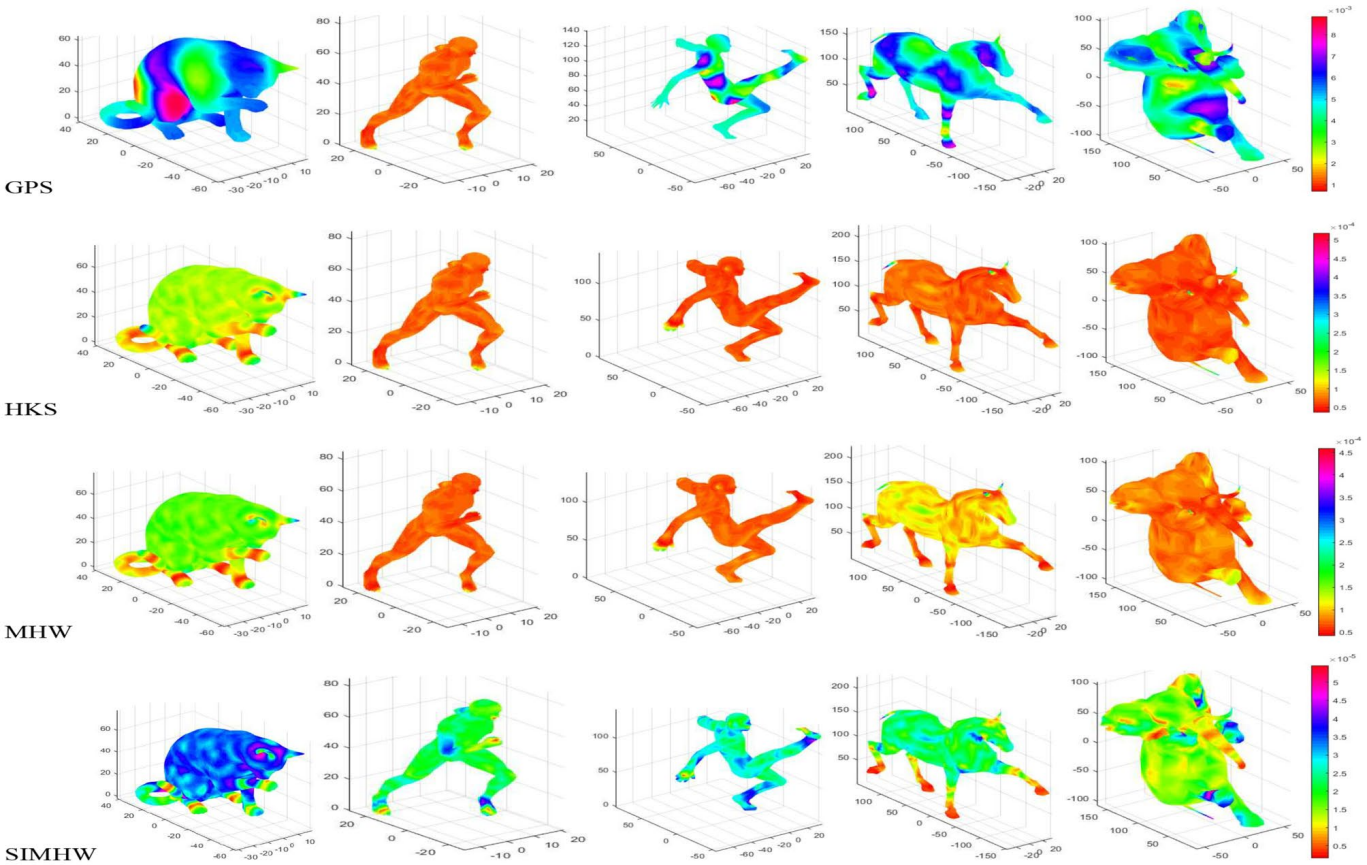
Description performance comparison of GPS, HKS, MHW and SIMHW.

To investigate and evaluate the efficiency of the SIMHW descriptor, we give the feature comparison results with the spectral descriptors GPS, HKS and MHW, as shown in Fig. 4. GPS is a global descriptor that describes the global information of a model. Compared with SIMHW, there has phenomenons of feature switching and misrepresentation in the symmetrical part of the model. For instance, the legs of the sports male and the forefoot of the horse should maintain the same feature color value, but there are relatively different color representations, which has a great influence on the similarity calculation of the model. Compared with HKS and MHW spectral descriptors, the SIMHW descriptor can accurately depict the local details of the model, can more effectively separate different regions of the shape, can describe and segment the limbs, trunk, tail, joints and ends of the model differently, and has stronger sensitivity and more effective recognition ability for regions with obvious curvature changes in non-rigid models.

### Robustness of the SIMHW descriptor

**Figure 5:**
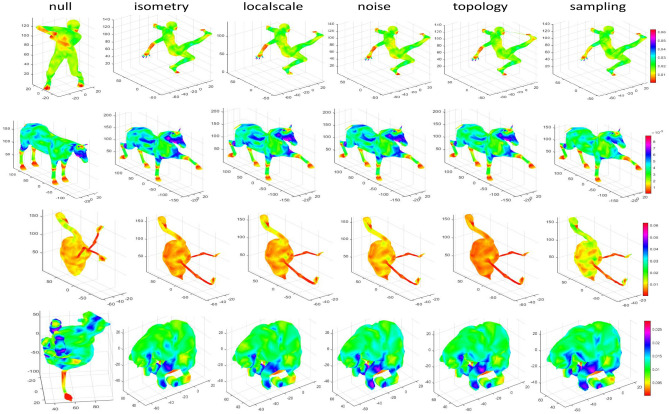
Robustness of SIMHW based on SHREC2010 and TOSCA dataset.

Based on TOSCA and SHREC2010 datasets, four models of isometric, localscale, noise, topology and sampling transformations are selected. The robustness of the SIMHW descriptor are shown in Fig. 5. Compared with the model without any non-rigid transformation(null), the SIMHW color value is almost consistent to other transformation poses, which can well describe the local or global information of the shape and have strong robustness to various non-rigid transformations with different intensities. Although the robustness of the crane model to the sampling transformation is weakened, but, the error of the corresponding feature color value is very small, and it can still be considered to be robust to the sampling transformation within a certain threshold.

**Figure 6:**
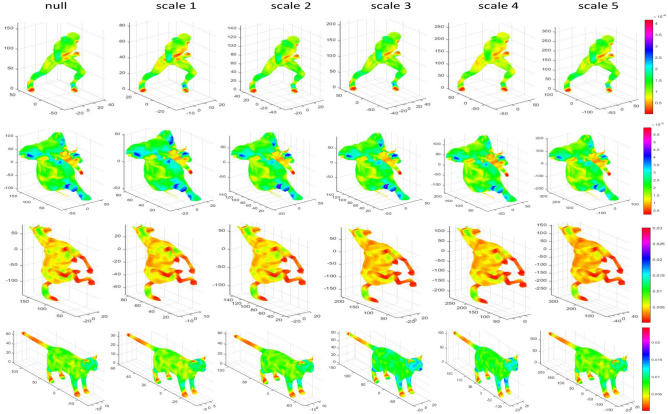
Robustness of SIMHW to scale transformation based on the SHREC2010 dataset.

Using the SHREC2010 dataset, the robustness of SIMHW to scale transformation is further proved. The SIMHW feature mapping of different models at five different scale transformation intensities is shown in Fig. 6. As we can seen that the SIMHW descriptor remains unchanged for scale transformations of different intensities, and the same shape has the same color value. The SIMHW descriptor can well describe the local or global attributes of the shape, especially for the regions where the curvature changes and the features are obvious, and the color values are displayed in warm colors. The closer the shape color is to red, the richer the feature information of the shape is, which is more favorable for effective recognition and matching between different shapes.

### Shape distance calculation

In order to more accurately measure and compare the effect of spectral descriptors under MHD distance calculation method, the Dunn validity index(DVI)^40^ coefficient is selected to evaluate the classification accuracy on the TOSCA, SHREC2010 and SHREC2015 datasets. The DVI coefficient describes the minimum distance between any two clusters divided by the maximum distance between any two points in the cluster. When measuring shape similarity, the larger the DVI coefficient, the smaller the intra-class distance, the larger the inter-class distance, and the better the separation between classes. Therefore, the similarity measure method with larger DVI coefficient should be selected as far as possible.

12 $$\begin{aligned} DVI=\frac{{\min \limits _{0<m\ne n<k}}{\{{{\min \limits _{\forall x_{i}\epsilon X_{m},x_{j}\epsilon X_{n}}}}\{\Vert x_{i}-x_{j} \Vert \}}\}}{\max \limits _{0<m\le k}{{\max \limits _{\forall x_{i},x_{j}\epsilon X_{m}}\{\Vert x_{i}-x_{j} \Vert \}}}} \end{aligned}$$

$X_{m}$ and $X_{n}$ represent the *m-*th and *n-*th cluster in the shape dataset, and *k* is the number of clusters. Table 1 shows the classification accuracy DVI values of the spectral operator on the datasets. The results show that the SIMHW descriptor can effectively recognize and classify similar shapes without the influence of scale transformation, and has the highest classification accuracy on three different non-rigid datasets. Compared with MHW, the DVI coefficients of SIMHW in TOSCA, SHREC2010 and SHREC2015 datasets increased by 18.2%, 15.5% and 7.5%, respectively.

**Table 1:** Shape classification accuracy calculated by MHD distance, the best results are displayed in bold.

Dateset	GPS	HKS	MHW	SIMHW
TOSCA	0.012 5	0.014 8	0.013 0	**0.031 2**
SHREC2010	0.004 5	0.000 9	0.002 3	**0.017 8**
SHREC2015	0.002 2	2.3511e−5	0.000 2	**0.007 7**

**Table 2:** Matching results using SIMHW descriptor based on TOSCA, SHREC2010 and SHREC2015 datasets.

	Class1	Class2	Class3	Class4	Class5	Class6
TOSCA						
Class1	**0.007 2**	6.705 2	1.109 5	0.055 2	6.986 8	2.872 0
Class2	6.705 2	**0.003 3**	1.385 2	4.067 0	0.041 7	0.286 2
Class3	1.109 5	1.385 2	**0.005 4**	0.513 5	2.778 8	0.461 8
Class4	0.055 2	4.067 0	0.513 5	**0.023 6**	5.665 2	2.292 3
Class5	6.986 8	0.041 7	2.778 8	5.665 2	**0.001 6**	1.149 5
Class6	2.872 0	0.286 2	0.461 8	2.292 3	1.149 5	**0.029 8**
SHREC2010						
Class1	**0.000 6**	**0.000 8**	0.000 9	0.011 0	0.031 5	0.001 0
Class2	0.000 8	0.001 0	0.001 1	0.013 3	0.034 1	0.001 4
Class3	0.000 9	0.001 1	**0.000 4**	0.014 2	0.035 6	0.001 8
Class4	0.011 0	0.013 3	0.014 2	**0.000 5**	0.002 8	0.006 4
Class5	0.031 5	0.034 1	0.035 6	0.002 8	**0.000 2**	0.022 3
Class6	0.001 0	0.001 4	0.001 8	0.006 4	0.022 3	**0.000 8**
SHREC2015						
Class1	**0.004 6**	0.005 4	**0.027 2**	0.007 3	0.023 2	0.103 5
Class2	0.005 4	**0.001 3**	0.043 9	**0.003 7**	0.025 8	0.122 3
Class3	0.027 2	0.043 9	0.030 0	0.050 9	0.090 0	0.181 0
Class4	0.007 3	0.003 7	0.050 9	0.004 0	0.025 0	0.108 4
Class5	0.023 2	0.025 8	0.090 0	0.025 0	**0.002 7**	0.050 3
Class6	0.103 5	0.122 3	0.181 0	0.108 4	0.050 3	**5.810 8e-8**

**Figure 7:**
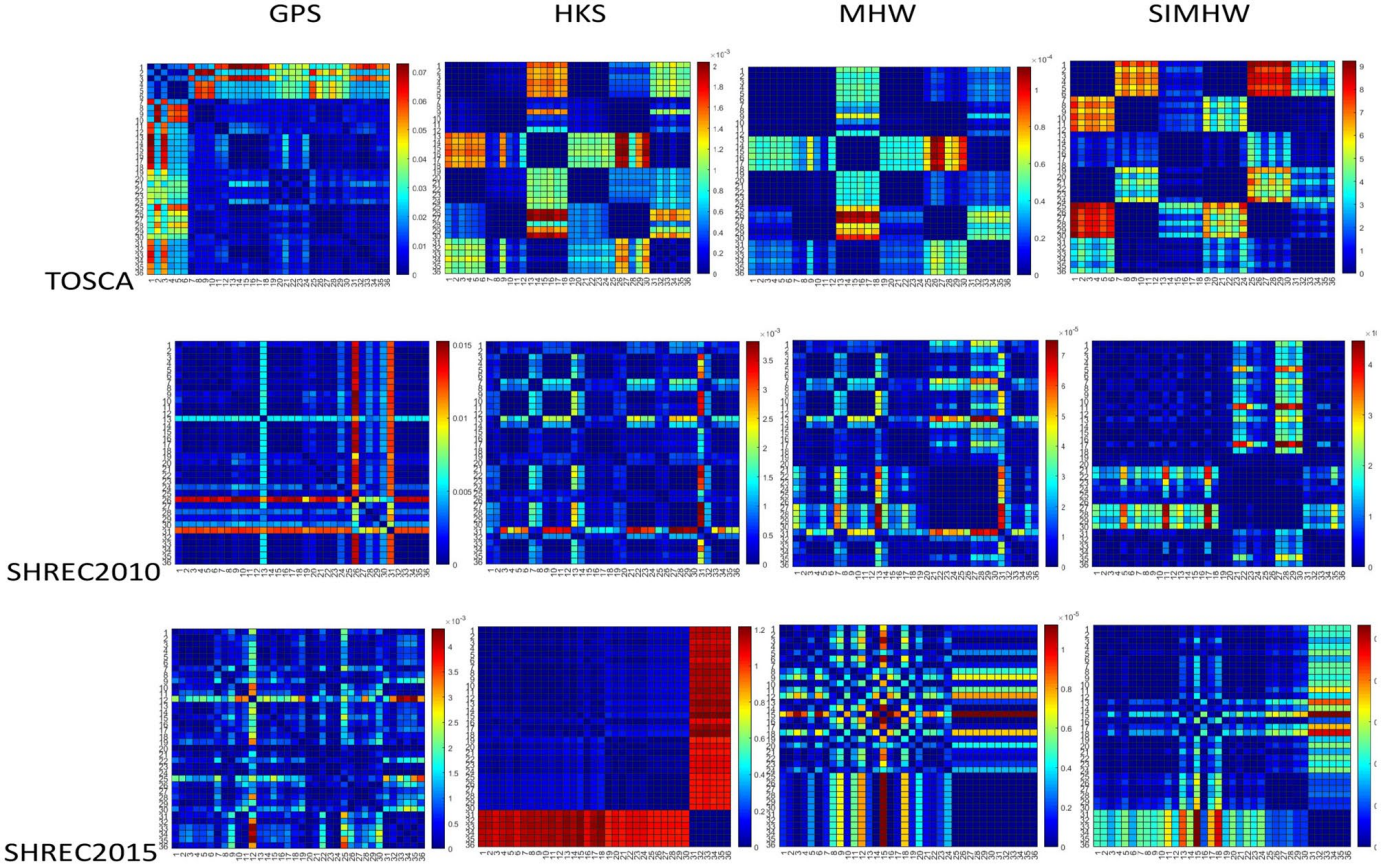
Visual thermodynamic diagram comparison results based on MHD distance matrix.

Six different classes of 36 models were selected from the TOSCA, SHREC2010 and SHREC2015 datasets, respectively. Table 2 shows the matching results using the SIMHW descriptor. We compare each object with all other objects in the TOSCA, SHREC2010 and SHREC2015 datasets. Each cell displays the average similarity measure *MHD*(*M*, *N*) between the two objects, where the minimum value (marked in bold) corresponds to the distance between the correctly matched objects, and the smaller the distance value, the better the match between the models. According to the calculated results of MHD distance, the thermodynamic diagram of shape distance classification is shown in Fig. 7. Cause the TOSCA dataset only includes the isometric transformation models, the deformation type is relatively single, and the distance between classes is quite different in the distance calculation, and the classification effect is generally good. Compared with other spectral descriptors, the distance value of the bold mark is the smallest at the diagonal position and has the highest matching degree, which indicates that SIMHW can correctly identify each model and achieve accurate classification.

SHREC2010 and SHREC2015 datasets contain various non-rigid transformation models, which are more challenging for the robustness and effectiveness of descriptors. Combined with classification accuracy DVI, distance thermodynamic diagram and matching results, we can see that although the difference in distance calculation results between models is not as significant as that of TOSCA dataset, the SIMHW still has better classification effect and matching results than other spectral descriptors. In the SHREC2010 dataset, GPS, HKS and MHW descriptors can only correctly identify some models, while SIMHW only has fuzzy classification in the first and second classes of models, and other categories have achieved correct classification. In the SHREC2015 dataset, GPS and MHW descriptors can only accurately match the first and sixth classes of models, HKS can only realize the rough matching of the fourth, fifth and sixth classes, and the discrimination between other categories is not obvious, but the SIMHW descriptor can almost effectively classify all categories correctly. In Table 2, there are inconsistencies between the minimum distance value (boldface) and the real category of SIMHW (diagonal position), especially on the SHREC2015 dataset, the main reason for this is that the SHREC2015 dataset contains some partial missing and hole models (especially the third class), which affects the stability of similarity calculation between models.

### Shape retrieval

According to the calculation results of 3D shape distance, the appropriate retrieval threshold is set to quickly query and extract the relevant shape, so the result of shape distance calculation determines the performance of shape retrieval. In order to verify the retrieval performance of the operator under non-rigid transformation postures, we give the shape retrieval results of GPS, HKS, MHW and SIMHW descriptors based on MHD distance calculation method on SHREC2010 and SHREC2015 datasets.

**Figure 8:**
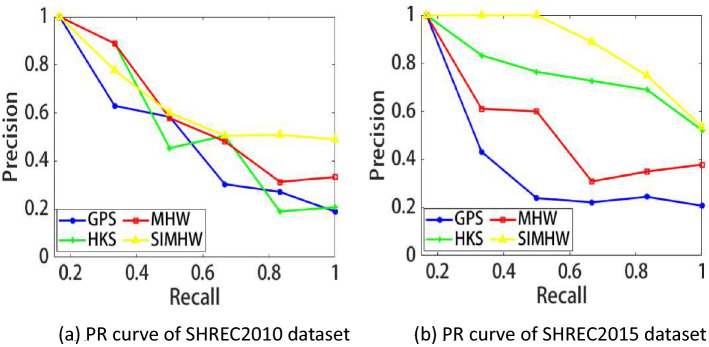
PR curve of the GPS, HKS, MHW and SIMHW descriptors.

Shape retrieval performance usually uses the PR curve (Fig. 8) to represent the relationship between retrieval accuracy (P) and retrieval recall (R). The retrieval accuracy refers to the ratio of the number of retrieved related shapes to the total number of shapes in the retrieval returned results, and the retrieval recall refers to the ratio of the number of retrieved related shapes to the total number of existing related shapes that can be ideally retrieved. To compare the retrieval performance in detail, we also used the retrieval numerical indicators, such as NN, FT, ST, E-M, $F_{2}-M$, Fall-out and MAP (Table 3), in which the mean average precision (MAP) can be used as a single-valued quantization representation the retrieval performance of the algorithm.

Table 3 shows the numerical indicators of shape retrieval using GPS, HKS, MHW and SIMHW on SHREC2010 and SHREC2015 datasets. Our method shows that the SIMHW descriptor is improved under different numerical metrics. Compared with GPS, HKS and MHW, the SIMHW method obtained better results on SHREC2010 dataset. SIMHW outperforms the MHW approach by 8.33% in NN, 10% in FT, 13.42% in ST, 3.81% in E-M, 4.55% in $F_{2}-M$, 1.66% in Fall-out and 4.73% in MAP. Compared with GPS and HKS, the values of the MAP were increased by 15.02% and 10.58%, respectively. Based on the SHREC2015 dataset, the value of SIMHW is higher than that on other algorithms, the SIMHW descriptor obtains almost good retrieval values(e.g., NN = 77.78%, FT = 83.33%, ST = 92.42%, E–M = 73.33%, $F_{2}-M$ = 68.75%, Fall-out = 4.44% and MAP = 86.24%). This method is not only obviously superior to all other methods, but also obtains better retrieval accuracy than the best method. It can also be seen from the table that compared with SHREC2010, the retrieval indicator of the SHREC2015 dataset is relatively high (MAP of SIMHW is 64.66% and 86.24%, respectively), indicating that in the retrieval ranking based on the SIMHW descriptor, the SHREC2015 dataset has a large number of models with similar shapes are queried, and the effective and correct retrieval of the model ranking is relatively high within the retrieval threshold range.

**Table 3:** Retrieval values based on the SHREC2010 and SHREC2015 datasets.

Dateset	SHREC2010				SHREC2015			
Index	GPS	HKS	MHW	SIMHW	GPS	HKS	MHW	SIMHW
NN	0.388 9	0.500 0	0.527 8	0.611 1	0.222 2	0.555 6	0.210 5	0.777 8
FT	0.466 7	0.600 0	0.633 3	0.733 3	0.266 7	0.666 7	0.533 3	0.833 3
ST	0.521 9	0.673 2	0.705 4	0.839 6	0.414 7	0.766 0	0.602 5	0.924 2
E–M	0.400 0	0.466 7	0.533 3	0.571 4	0.266 7	0.577 8	0.466 7	0.733 3
$F_{2}-M$	0.375 0	0.437 5	0.500 0	0.545 5	0.250 0	0.575 0	0.437 5	0.687 5
Fall-out	0.122 2	0.100 0	0.094 4	0.077 8	0.155 6	0.088 9	0.111 1	0.044 4
MAP	0.496 4	0.540 8	0.599 3	0.646 6	0.391 2	0.693 5	0.541 6	0.862 4

The PR curves of SHREC2010 and SHREC2015 datasets are shown in Fig. 8. The closer the upper right corner of the PR curve is to 1, the higher the retrieval accuracy is on the basis of ensuring a certain recall rate, the SIMHW descriptor is represented by a yellow curve. It can be seen from the figure that the overall retrieval performance of SHREC2015 dataset is better than that of SHREC2010 dataset, which is also consistent with the retrieval numerical calculation results. On the SHREC2010 dataset, when the recall rate is greater than 0.48, the SIMHW curve gradually shows higher retrieval accuracy than other methods. On the SHREC2015 dataset, the yellow curve can almost completely contain other spectral descriptor curves, indicating that the retrieval accuracy of the SIMHW descriptor has been higher than other spectral descriptors as the recall rate increases. Both datasets contain scale transformation models, cause the MHW descriptor is more sensitive to scale transformation, especially on the SHREC2015 dataset, the retrieval performance is worse than HKS. Combining the two datasets, from the retrieval indicators and PR curves, the retrieval efficiency of SIMHW is significantly higher than that of MHW descriptor, indicating that the proposed method is effective for the recognition and classification of non-rigid transformations and can better realize shape retrieval.

**Figure 9:**
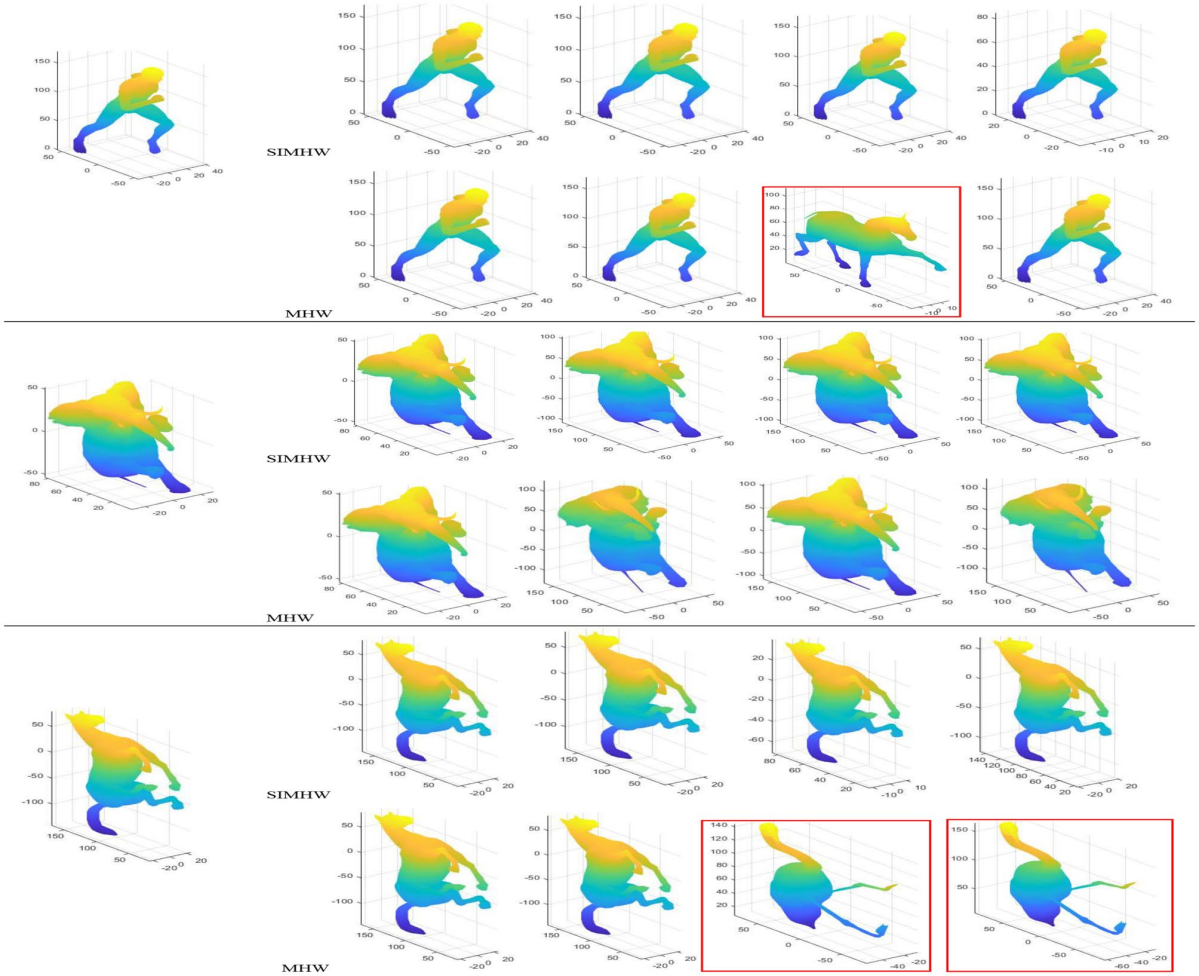
Shape retrieval results (SHREC2010). Left: queries; right: first matches using SIMHW and MHW.

**Figure 10:**
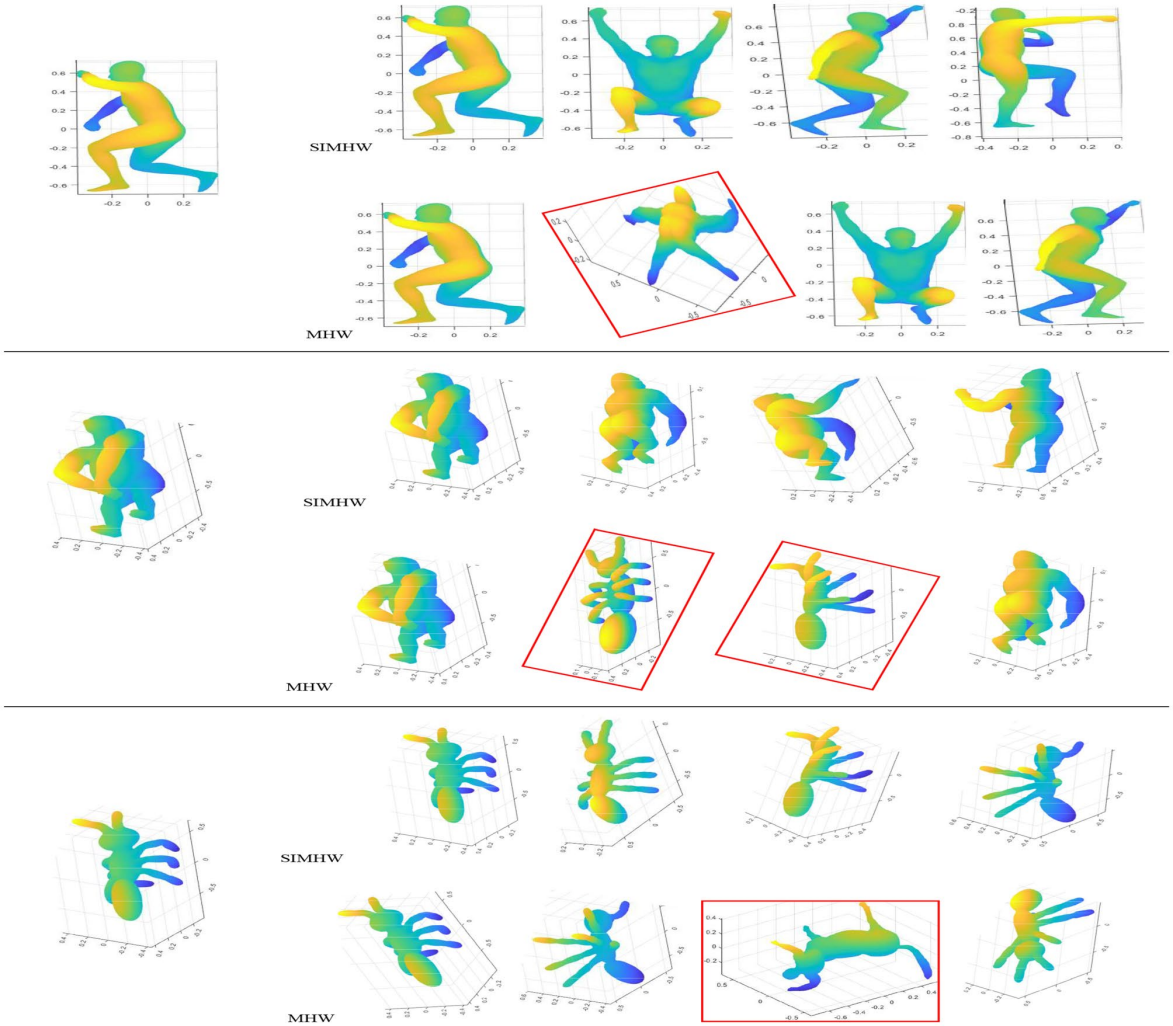
Shape retrieval results (SHREC2015). Left: queries; right: first matches using SIMHW and MHW.

Figures 9 and 10 shown the TOP4 matching models retrieved using MHW and SIMHW descriptors. The retrieval results of some models based on the SHREC2010 dataset are shown in Fig. 9, the SIMHW descriptor is robust to scale transformation and can effectively represent non-rigid shape features. Even if there has localscale model, it can produce correct matching results that satisfy the retrieval threshold. When MHW is used for retrieval, the sports male model and the horse model have mismatched in the leg feature region, and misclassification was also generated by feature information between the horse and Crane models. Figure 10 shown the TOP4 retrieval results on the SHREC2015 dataset. From the figure, we can seen that the SIMHW descriptor can match correctly, query the similar models, and have strong recognition and classification capabilities for different deformation models. However, when the retrieval experiment is based on the MHW descriptor, the three groups of query models have the phenomenon of misclassification, and the misclassification model ranked first. Since the MHW descriptor does not have the invariance of scale transformation, it affects the feature representation ability of the operator, and causes difficulties and disturbances in the computation of similarity between models.

## Discussion

The shape descriptor is invariant to scale transformation and play an important role in 3D shape analysis. This invariance can greatly reduce the pre-processing process of deformation shapes, and can extract and calculate the features of shape directly from the original data. In this study, we propose a scale-invariant Mexican Hat wavelet (SIMHW) as a local spectral shape descriptor which has good feature description performance and stable invariance, it can describe the shape more precisely without losing the shape information. Using the inherent advantages of the LB operator, SIMHW is more versatile for different forms of models (mesh and point cloud). It can retain the geometric and intrinsic properties of the model, and can better describe the details of the shape.

We randomly select six classes of shapes on the SHREC2010 dataset, each of which contains six non-rigid transformation models (isometric, noise, sampling, scale, localscale and topology). In order to analyze and compare with the recent non-rigid retrieval datasets, based on the same selection principle, we also selected 36 models with different non-rigid transformation poses on the SHREC2015 standard retrieval dataset. The robust performance of the shape descriptor has been analyzed in the experiment, and the results show that the SIMHW algorithm is less affected by the data selection and can select any model, but in order to verify the scale-invariant performance of SIMHW effectively, the scale transformation model must be placed in the datasets. The SIMHW value corresponding to the twelfth component $\omega _{12}$ is used as the local descriptor of feature description and distance calculation. Since HKS, MHW and SIMHW are all feature sequences, it is difficult to balance the relationship between description accuracy and computational complexity. In order to fairly compare the classification effect and retrieval performance of the algorithm for the scale transformation model, we refer to the experimental parameter settings of Sun^16^ and select the same parameters as HKS.

The robustness and effectiveness of the SIMHW descriptor is evaluated through non-rigid 3D datasets, and the performance is analyzed and compared through similarity calculation and shape retrieval experiments. The experimental results based on TOSCA, SHREC2010 and SHREC2015 datasets show that the SIMHW descriptor has invariance with scale, localscale and isometric transformations, and is robust to topology, noise and sampling transformations. Compared with MHW, the SIMHW descriptor is more stable for various deformed shapes, can effectively measure the distance between shapes, and can better realize accurate matching and shape retrieval.

The method proposed in this paper is a non-learning method based on the Riemann framework, which has low computational complexity and is easy to understand. Aiming at the multi-time parameter characteristics of HKS and SIHKS, we want to construct a more compact and efficient shape descriptor, which can better balance the accuracy and computational complexity of shape description. With the rapid development of geometric deep learning technology, the combination of spectral analysis and geometric deep learning through learning methods on large sample datasets can obtain more refined and differentiated feature information of the models, which is also a problem worthy of mining and research.

## Data Availability

The datasets used during the current study available from the corresponding author on reasonable request.
